# Genetically diagnosed Birt–Hogg–Dubé syndrome and familial cerebral cavernous malformations in the same individual: a case report

**DOI:** 10.1007/s10689-016-9928-y

**Published:** 2016-10-08

**Authors:** James Whitworth, Brian Stausbøl-Grøn, Anne-Bine Skytte

**Affiliations:** 1 Department of Medical Genetics, University of Cambridge, Cambridge, UK; 2 Department of Radiology, Aarhus University Hospital, Aarhus, Denmark; 3 Department of Clinical Genetics, Aarhus University Hospital, Aarhus, Denmark

**Keywords:** Double heterozygote, Birt–Hogg–Dubé syndrome, Cerebral cavernous malformation, Genetics

## Abstract

When faced with an unusual clinical feature in a patient with a Mendelian disorder, the clinician may entertain the possibilities of either the feature representing a novel manifestation of that disorder or the co-existence of a different inherited condition. Here we describe an individual with a submandibular oncocytoma, pulmonary bullae and renal cysts as well as multiple cerebral cavernous malformations and haemangiomas. Genetic investigations revealed constitutional mutations in *FLCN*, associated with Birt–Hogg–Dubé syndrome (BHD) and *CCM2*, associated with familial cerebral cavernous malformation. Intracranial vascular pathologies (but not cerebral cavernous malformation) have recently been described in a number of individuals with BHD (Kapoor et al. in Fam Cancer 14:595–597, 10.1007/s10689-015-9807-y, 2015) but it is not yet clear whether they represent a genuine part of that conditions’ phenotypic spectrum. We suggest that in such instances of potentially novel clinical features, more extensive genetic testing to consider co-existing conditions should be considered where available. The increased use of next generation sequencing applications in diagnostic settings is likely to lead more cases such as this being revealed.

## Introduction

Birt–Hogg–Dubé syndrome (BHD) is a rare autosomal dominant inherited neoplasia syndrome. As with most such disorders, it is characterized by variable expression and incomplete penetrance. First described in 1977 in a family with facial fibrofolliculomas (the most prevalent manifestation of the disease) it was subsequently recognized that affected individuals also have an increased risk of pulmonary cysts, spontaneous pneumothoraces and renal tumours, particularly oncocytoma and chromophobe renal cell carcinoma. A variety of other features have been described including thyroid nodules and salivary gland oncocytoma [2].

Genes associated with inherited cancer syndromes are often discovered through the study of individuals affected with a particular tumour type. However, the range of tumours associated with mutations in a particular gene may expand as more cases are uncovered through research or clinical testing. Recently it has been proposed that intracranial vascular pathologies may also form part of this condition following the report of three BHD patients with a saccular aneurysm, an arteriovenous malformation and a carotid artery aneurysm [1].

BHD is caused by constitutional mutations in *FLCN*, a tumour suppressor gene that encodes the protein folliculin. The exact function of this protein is still under evaluation but currently it is known to be involved in the regulation of the mTOR pathway [3]. One consequence of upregulation of the mTOR pathway is increased HIF1-α activity [4], which has been observed in a patient derived renal tumour cell line null for *FLCN* gene product [5]. It has therefore been proposed (given the role of HIF1-α in angiogenesis) as a mechanism leading to an elevated risk of intracranial vascular pathology in patients harbouring *FLCN* mutations [1].

The definition of a rare disease is a frequency of less than 1 in 2000 [6] but there is an appreciable probability of a person harbouring two diseases caused by separate genetic aberrations by chance. In theory, these individuals are more likely (relative to those with only one condition) to be referred for genetic counselling because of a striking medical history but a number phenotypic consequences arising from this situation are possible.

The clinician presented with a patient who shows features of more than one inherited syndrome could entertain a number of possibilities. These include a single condition with other features in the patient due to non-genetic causes, a new clinical feature of a recognised condition or indeed, two genetic conditions.

Here we present a case of BHD syndrome and hereditary cerebral cavernous malformations due to the combination of pathogenic variants in both *FLCN* and *CCM2* in the same individual. The case highlights that intracranial vascular pathologies may be a manifestation of concurrent mutations in other genes rather than being caused by *FLCN* mutations alone.

## Case report

**Fig. 1:**
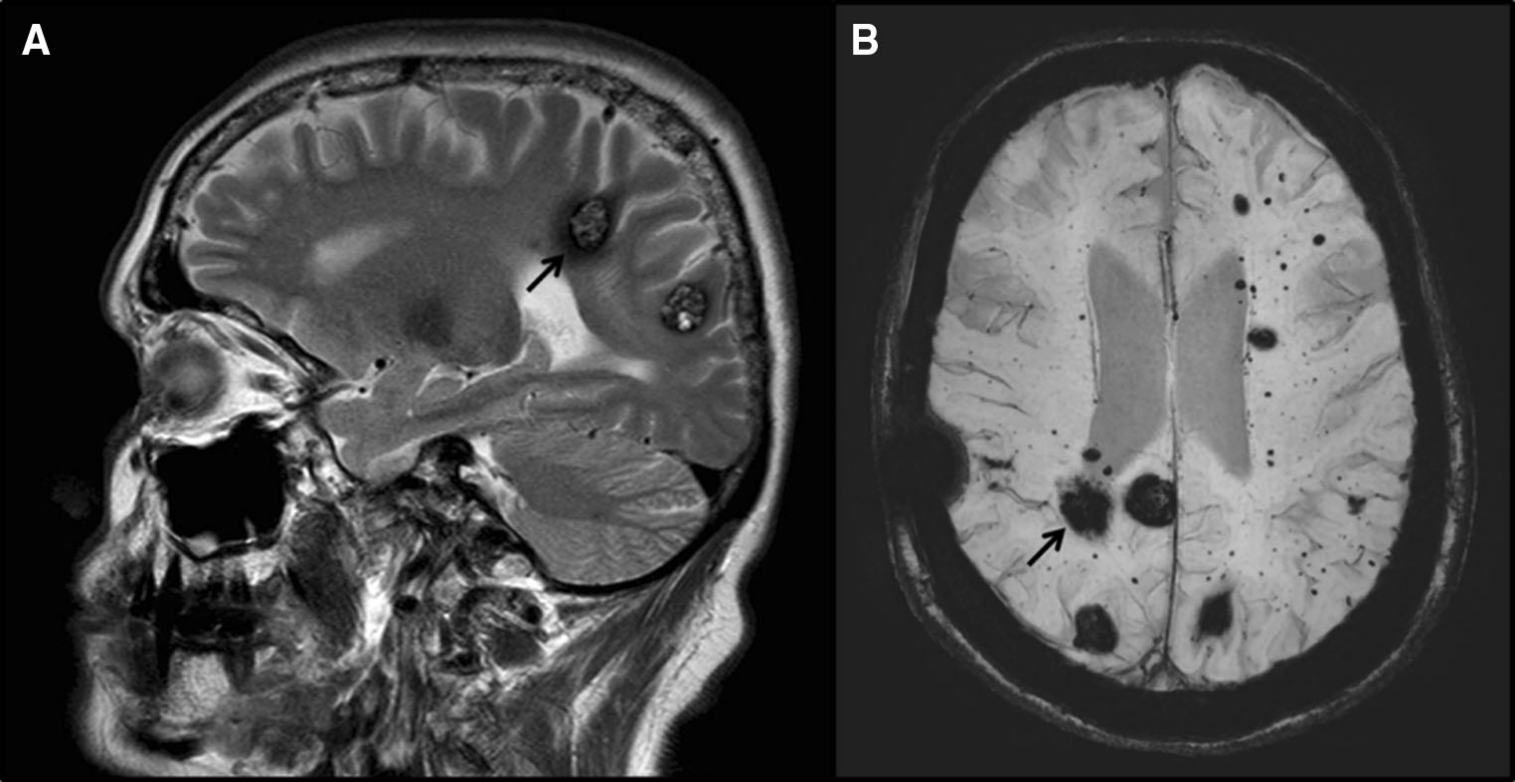
Cerebral magnetic resonance imaging scan of proband showing multiple haemangiomas and cavernous malformations

A 58-year-old woman was referred to clinical genetics following the diagnosis of a variety of disease manifestations. A mandibular swelling had recently been detected, biopsy of which showed an oncocytoma in the submandibular gland. She had previously consulted the neurosurgical team due to the presence of multiple hemangiomas/cavernous malformations in the cerebrum and cerebellum, first diagnosed at the age of 48 years (Fig. 1). Interrogation of her medical history revealed that she had been diagnosed with pulmonary bullae at the age of 35 years following a spontaneous pneumothorax in the weeks after the birth of her fourth child. At 48 years she had also developed a goitre and facial rash.

**Fig. 2:**
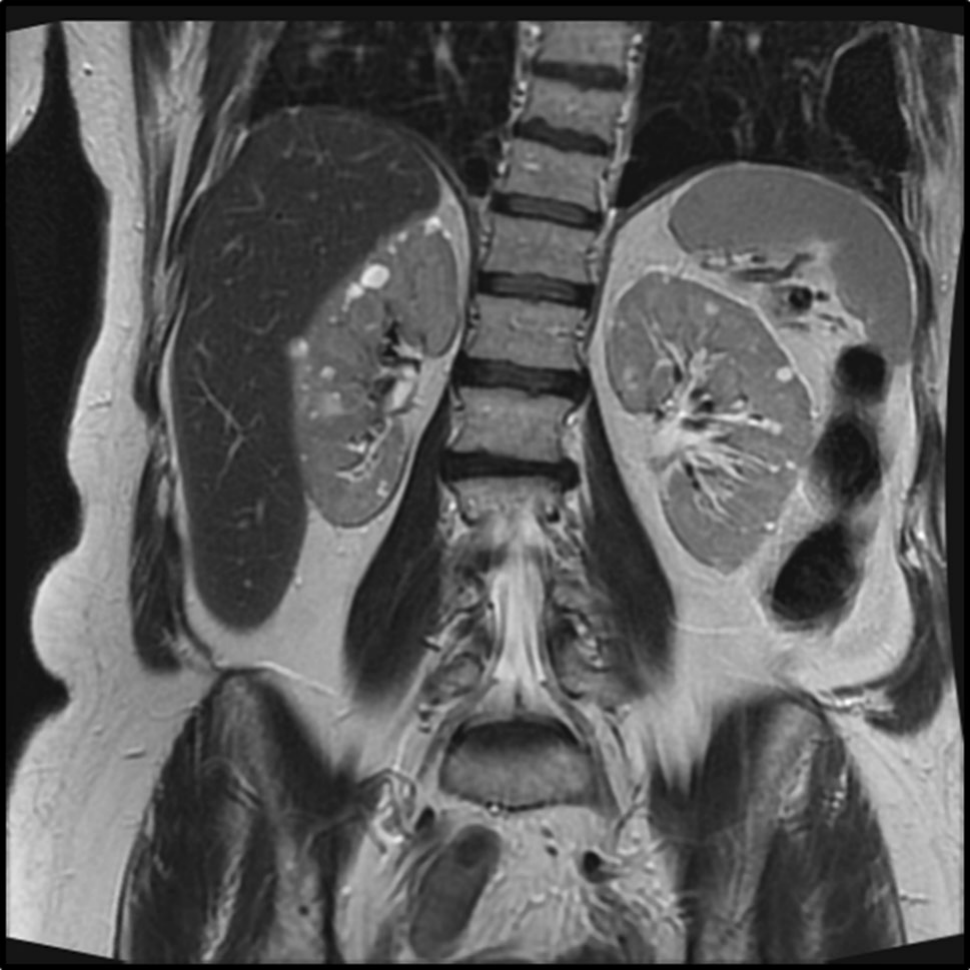
Abdominal magnetic resonance imaging scan of probands showing multiple renal cysts and pulmonary bullae

From the medical history and clinical appearance, two rare diseases were considered; BHD and hereditary cavernous cerebral malformations (CCM). Genetic investigations of the relevant genes using a single candidate gene testing strategy (*FLCN, KRIT1, CCM2* and *PDCD10*) revealed the two heterozygous frameshift mutations (*FLCN* c.158del, p.Gln53 fs and *CCM2* c.319C > T, p.Gln107*). Follow-up scan of the kidneys revealed multiple small cysts and pulmonary bullae (Fig. 2).

The family history was only partially complete as the patient had been adopted as an infant. There was therefore no knowledge of the paternal side of the family. Her mother died of unknown reasons at 59 years old and she had a half-brother on that side of the family who was diagnosed with an intra-cerebral haematoma at age 26 years. That individual was identified by genetic testing as harbouring the familial *CCM2* mutation but not the *FLCN* mutation. All of her four children are reported to be healthy, although the third child was observed to have a facial rash similar to that of the proband.

## Discussion

Cerebral cavernous malformations consist of immature dilated capillaries without intervening brain tissue. They occur in both a sporadic and familial context with three genes (*KRIT1*, *CCM2* and *PDCD10*) currently implicated in the causation of the latter. Familial CCM is inherited in an autosomal dominant manner and typically presents in early to mid-adulthood, often with multiple lesions that may cause headaches, neurological deficits, seizures and cerebral haemorrhages [7].

CCMs have an appreciable prevalence in the general population at around 1/200 [8] so may well occur by chance in patients with genetic conditions. However, the presence of multiple CCMs and a truncating *CCM2* mutation in this individual provides compelling evidence that the genetic variant is the cause. Although there is a possible mechanistic link between *FLCN* mutations and vascular lesions via HIF1-α dysregulation and intracranial vascular pathologies have been reported in BHD patients, no CCMs were reported in that series suggesting BHD is unlikely to be causative in this patient.

The co-occurrence of *FLCN* and *CCM2* mutations appears improbable given that both associated conditions are considered rare with only a small number of families being described. However, prevalence of genetic conditions may be underestimated due to incomplete penetrance and low rates of testing of the relevant gene by clinical services. The increasing use of massively parallel sequencing in clinical practice in the form of gene panels and exome sequencing should go some way to address these issues and produce a more accurate prevalence estimate. Ultimately, mutations combinations such as this may start to appear less improbable as a result.

Once the presence of multiple pathogenic mutations has been detected in an individual, a pertinent question is the likely phenotypic effect of that combination compared to if either was present in isolation. In this case the patient presented with a phenotype consistent with the concurrent diseases acting independently. However, it is feasible that multiple mutations may act synergistically to produce more severe or novel phenotypes or even attenuate severity through mechanisms such as synthetic lethality. Again, increased detection of cases through application of next generation sequencing technologies (and sharing in public databases) should lead to better assessments of the likely effects of particular combinations. Intriguingly, in the genetics department where this patient consulted, 12 families with BHD syndrome are known to the clinical team. Of these, three contain at least one individual with another rare inherited condition. This may reflect an underestimate of the rarity of *FLCN* mutations or perhaps even a higher biological tolerance for multiple mutations involving this gene than others.
